# CuFe_2_O_4_/Polyaniline (PANI) Nanocomposite for the Hazard Mercuric Ion Removal: Synthesis, Characterization, and Adsorption Properties Study

**DOI:** 10.3390/molecules25122721

**Published:** 2020-06-12

**Authors:** Saad S. M. Hassan, Ayman H. Kamel, Amr A. Hassan, Abd El-Galil E. Amr, Heba Abd El-Naby, Mohamed A. Al-Omar, Ahmed Y. A. Sayed

**Affiliations:** 1 Chemistry Department, Faculty of Science, Ain Shams University, Abbasia, Cairo 11566, Egypt; amr_hassan@sci.asu.edu.eg (A.A.H.); hoba_science@hotmail.com (H.A.E.-N.); 2 Department of Chemistry, Virginia Commonwealth University, Richmond, VA 23284, USA; 3 Pharmaceutical Chemistry Department, Drug Exploration & Development Chair (DEDC), College of Pharmacy, King Saud University, Riyadh 11451, Saudi Arabia; malomar1@ksu.edu.sa (M.A.A.-O.); ahmedyahia009@gmail.com (A.Y.A.S.); 4 Applied Organic Chemistry Department, National Research Center, Dokki, Giza 12622, Egypt

**Keywords:** CuFe_2_O_4_ nano-particles, CuFe_2_O_4_/PANI composite, mercury (II) removal, adsorption

## Abstract

Copper ferrite nano-particles (CuFe_2_O_4_) were synthesized, characterized, modified with polyaniline to form CuFe_2_O_4_/PANI nano-composite. They were used as new adsorbents for the removal of the hazardous mercuric ions from aqueous solutions. High resolution transmission electron microscope (HR-TEM), X-ray diffraction (XRD), Fourier-transform infrared (FT-IR) and Brunauer–Emmett–Teller (BET) were used for the characterization of the synthesized CuFe_2_O_4_ nano-particles (NPs) in presence and absence of PANI nano-composite. The synthesized CuFe_2_O_4_NPs were of spherical shape with an average size of 10.8 nm. XRD analysis displayed crystal peaks for CuFe_2_O_4_NPs and amorphous peaks CuFe_2_O_4_/PANI nano-composite due to the existence of polyaniline layer. Contact time, adsorbent dose, solution pH, adsorption kinetics, adsorption isotherm and recyclability were studied. The method at the optimum conditions exhibited high performance with high mercury removal percentage of up to 99% with a maximum adsorption capacity 12.5 and 157.1 mg/g for CuFe_2_O_4_ and CuFe_2_O_4_/PANI, respectively. The adsorption processes were fitted to Langmuir isotherms. The adsorption behavior of CuFe_2_O_4_@PANI composite towards Hg^2+^ ions is attributed to the soft acid–soft base strong interaction between PANI and Hg(II) ions. High stability and enhanced re-usability are offered using CuFe_2_O_4_@PANI composite due to its enhanced removal efficiency. No significant removal decrease was noticed after five adsorption–desorption cycles. In addition, it possesses an easy removal from aqueous solutions by external magnetic field after adsorption experiments. These indicated the enhancement of polyaniline to the surface of CuFe_2_O_4_ toward the adsorption of mercury from aqueous solutions.

## 1. Introduction

A clean water resource is a vital and necessary goal for the whole world. Toxic heavy metals like Hg, Pb, Cd, and Ni are considered the most dangerous environmental pollutants in the water, thus becoming of prior anxiety because of their toxicity and non-biodegradability to plants, animals and human [1,2]. Mercury is one of these heavy metals that can cause serious environmental and health problems as chronic and acute poisoning. It exists in different forms such as metallic Hg, Hg^+^, Hg^2+^, and organic mercury containing phenyl, methyl, and ethyl groups, etc. It causes different diseases such as Alzheimer’s disease, amyotrophic lateral sclerosis, Parkinson’s disease, and damaging of the immune system and kidneys. Mercury is considered as prior hazardous pollutant by the Agency for Toxic Substances and Disease Registry [3]. One of its natural sources is the volcanoes that produce almost half of the mercury emissions released in atmosphere. It is also produced from different industrial sources such as pharmaceuticals, chloralkali, plastic, textile, paint, rubber, paper, cement, electronic industry, coal combustion, fertilizers, oil refining, and rubber processing [4,5]. The other half is generated by humans by various means including 65% in combustion, 11% in the production of gold, 6.8% in the production of non-ferrous metal, 6.4% in the production of cement, 3.0% in the waste disposal including municipal waste, and 3.0% in the production of caustic soda [6]. According to the World Health Organization (WHO), 1 µg/L is the maximum permissible concentration of Hg(II)in drinking water [7]. According to the European Union (EU), the maximum acceptable level of Hg(II) is 5 µg/L for wastewater discharge [8,9,10]. River and lake water in the nearby industries may contain mercuric discharges which are fatal for aquatic as well as for human life. These discharges could accumulate in the stomach and remain non digestible resulting in the formation of cancerous diseases. Long-term exposure to mercury could cause serious damage to nerves, brain, kidney, lung irritation, eye irritation, skin rashes, vomiting, and diarrhea [11].Researchers have been used a lot of techniques to get rid of heavy metals in particularly mercury ion from waste water such as sorption and filtration [12], ion exchange [13,14], chemical precipitation [6], adsorption [15,16,17], solid phase extraction [18], and adsorption process using nano-materials [19,20,21,22,23,24,25,26,27]. The adsorption technique is the most effective and commonly used due to its high removal efficiency and cheapness.

Recently, there is a focus on the application of nano-materials in the removal of different environmental pollutants. This is based on their distinctive properties such as high surface area, high adsorption, and special photoelectric property. However, they are suffering from difficulty of their separation from aqueous solutions due to their small particle size which restricts the application in water treatment. So, it is preferable using magnetic nano-materials that can be easily separated from solution with external magnetic field [28,29,30].

Magnetic nano-materials possess adsorbent properties that qualify them for use as promising adsorbent materials, which open up a wide field for engineering separation applications. These magnetic nano-particles can be separated based on their nanostructures due to the easy direction of magnetization, which will vary depending on the arrangement of the atoms in the magnetic structure [31,32,33]. Applying a low density magnetic field stimulates the magnetization of the material and therefore makes the use of magnetic force possible, but when the magnetic field is cut off, the magnetization immediately decreases to zero. This last point is important for the release of particles after adsorption of the waste [34,35]. The main drawback of using magnetic nano-particles is the low potential pollutant removal ability. To invade this defect, the surface of magnetic nano-particles has been modified. The surface properties of nano-particles can be greatly enhanced after this modification. This is preferred through the Van der Waals interaction between the modified material and the reduced solvent shielding of the ions in the interlamellar environment.

Polyaniline (PANI) has attracted much attention because of its several unique properties [36,37,38]. It is highly stable in air and soluble in various solvents and exhibits dramatic changes in its electronic structure and physical properties in the protonated state. It also shows magnetic behavior because of its high spin density [39,40].

In the present work, modification of CuFe_2_O_4_ nano-particles (NPs) with polyaniline was used as a novel adsorbent for mercury removal in aqueous solutions. The nano-particles were synthesized, characterized, and used as an adsorbent for mercury removal under optimum conditions. The removal efficiency of the prepared adsorbents was investigated, and their adsorption and desorption behaviors towards mercury species were studied.

## 2. Results and Discussion

### 2.1. Adsorbent Characterization

#### 2.1.1. X-ray Diffraction Pattern

**Figure 1:**
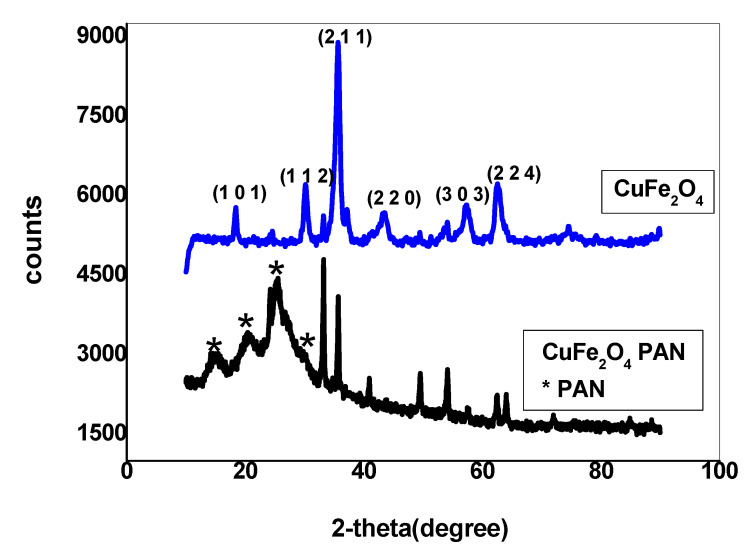
Pattern of both CuFe_2_O_4_ and CuFe_2_O_4_/PANI nano-composites.

The phase identification of CuFe_2_O_4_ and CuFe_2_O_4_/PANI nano-composites was illustrated by X-ray diffraction (XRD) as shown in Figure 1. All of the high intensity peaks are indexed and refined as tetragonal structure with I41/amd space group, which is consistent with standard Joint Committee on Powder Diffraction Standards (JCPDS) card no. 34-0425.The obtained XRD pattern exhibits good crystallinity for CuFe_2_O_4_. The reflection plans (101), (112), (211), (220), (303), and (224) coincide with the tetragonal spinel phase for CuFe_2_O_4_ with a characteristic peak appears at 2*θ* 35.5°. The reflection plans (010), (100), and (110) coincide with the amorphous phase of standard data for polyaniline. It is apparent that the broad diffraction peak centered at 2*θ* value 25.3° (110) in Figure 1 is the characteristic peak of the PANI layer. This can be ascribed to the periodicity parallel and perpendicular to the polymer chains, respectively [41]. The characteristic peak of CuFe_2_O_4_ still appears at 35.5° and little shift for the other peaks when doped with PANI. The average crystalline size of the prepared nano-composite was calculated using Scherrer’s equation [42]:

*d* = 0.9 *λ*/*β cos θ* (1)

where *d* is the average crystalline size, *λ* is the wavelength of CuK*α,β* is the full width at half maximum (FWHM) of most intense diffraction peak (211), and *θ* is the Bragg’s angle. The average particle size is estimated to be 10.8 and 23.4 nm for CuFe_2_O_4_ and CuFe_2_O_4_/PANI nano-composites, respectively.

#### 2.1.2. High Resolution Transmission Electron Microscopy (HRTEM)

**Figure 2:**
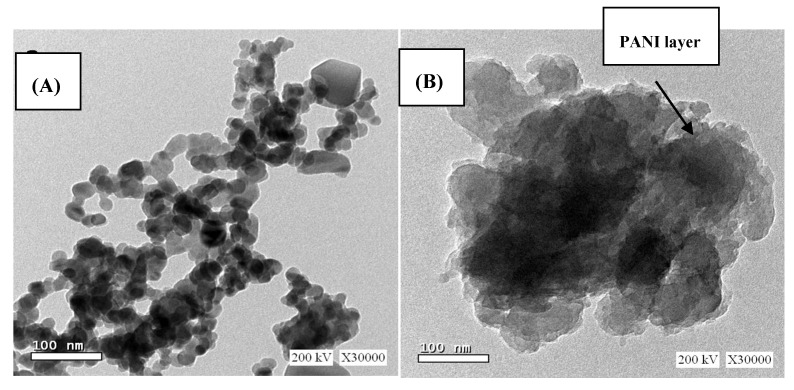
Images of (**A**) CuFe_2_O_4_ and (**B**) CuFe_2_O_4_/PANI nano-composites.

TEM images for CuFe_2_O_4_ nano-particles showed spherical shaped nano-particles with small agglomeration and nano sizes of 10.8 nm that coincides with the XRD result. The particles are dense and regularly distributed with clear boundary between neighboring particles as observed in Figure 2a. TEM images of CuFe_2_O_4_/PANI nano-composite revealed the light shell nature of PANI in which dark core copper ferrite particles are embedded as shown in Figure 2b.

#### 2.1.3. Brunauer–Emmett–Teller (BET)

**Figure 3:**
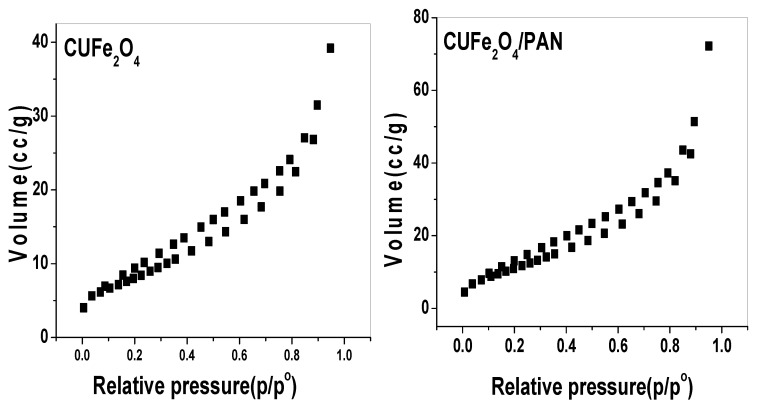
N_2_ adsorption–desorption isotherms of coupled CuFe_2_O_4_ and CuFe_2_O_4_/PANI (polyaniline) nano-composites.

The N_2_ adsorption–desorption experiment at 77 K for CuFe_2_O_4_ and CuFe_2_O_4_/PANI nano-composites are shown in Figure 3. The figure shows an adsorption isotherm of the type IV with a hysteresis loop that is associated with capillary condensation within the mesoporous regions [43], with a hysteresis loop type H3, which is usually indicative of aggregates of platelet particles or adsorbents containing slit pores. The initial part of the isotherm (until *p/p^o^* ≈ 0.4) can be attributed to monolayer/multilayer adsorption because it follows the same path of desorption, which demonstrates weak adsorbate–adsorbent interactions. The hysteresis loop begins at *p/p^o^* = 0.4 and it ends at *p/p^o^* = 0.95; the hysteresis loop exhibits limited adsorption. This phenomenon is related to the presence of particles that are not rigidly joined together. The BET surface area and pore volume of the nano-composite are recorded in Table 1. The pore size of CuFe_2_O_4_ nano-particles was about 9.9 nm. It was regarded as a mesoporous material of surface area 44.7 m^2^/g and pore volume of 0.11 cm^3^/g. For CuFe_2_O_4_/PANI nano-composites, the BET surface area is lower, around 30.8 m^2^/g, due to the lower cumulative volume of pores (0.06 cm^3^/g).

#### 2.1.4. Fourier Transforms–Infrared Spectroscopy (FTIR)

The FTIR spectra of CuFe_2_O_4_ and CuFe_2_O_4_/PANI nano-composites are shown as supplementary material in Supplementary Materials Figure S1. CuFe_2_O_4_ spectrum has only a characteristic peak at 574.9 cm^−1^ of M–O bond while CuFe_2_O_4_/PANI spectrum has several characteristics peaks corresponding to polyaniline. These include peaks at 3419.1 cm^−1^ assigned for N–H stretching, 1561.1 cm^−1^ assigned to stretching vibration of C=C, 1469.7 cm^−1^ assigned to stretching vibration of C–C, 1298.7 cm^−1^ C–N stretching vibrations, 1135.8 cm^−1^ for C–H bending mode and 777 cm^−1^ assigned to the wagging of =C–H. Hence the obtained results confirm the presence of copper ferrite nano-particles doped PANI.

#### 2.1.5. Thermal Analysis

Thermal-gravimetric analysis (TGA) of CuFe_2_O_4_/PANI is presented in Figure S2. It showed an overall weight loss of 35% in the range of 25–800 °C. A weight loss before 100 °C is noticed in the TGA curve due to residual water evaporation. Another weight loss is noticed within the ranging from 310 to 480 °C and 480 to 630 °C due to the thermal degradation of the lower and the higher weight PANI chains, respectively.

### 2.2. Adsorption Study

Nano-composite particles consisting of CuFe_2_O_4_ and that doped with PANI were prepared and tested as adsorbing substances to remove mercuric ions from aqueous solutions and some industrial waste water.

#### 2.2.1. Effect of Mercury Concentration

**Figure 4:**
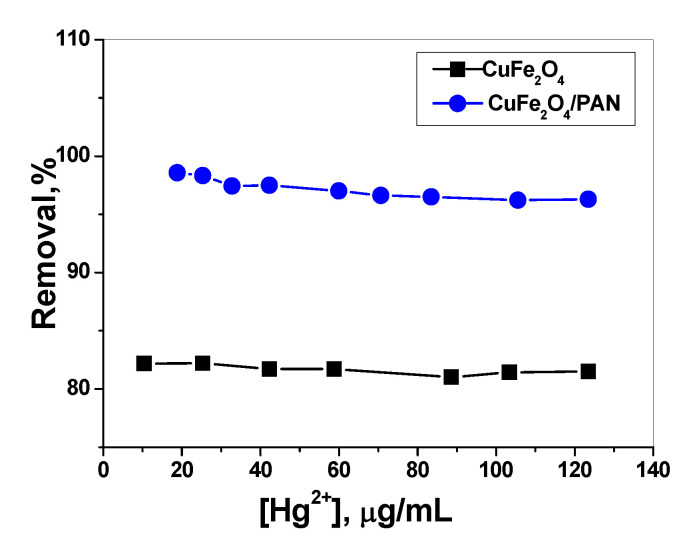
Effect of Hg^2+^ concentration on adsorption optimization using CuFe_2_O_4_ and CuFe_2_O_4_/PANI nano-composites sorbents; Conditions: (V = 30 mL, contact time = 2 h for CuFe_2_O_4_ and 1 h for CuFe_2_O_4_/PANI, adsorbent amount = 0.1 g CuFe_2_O_4_ and 0.2 g CuFe_2_O_4_/PANI and pH 7).

The removal efficiency of mercury ions using CuFe_2_O_4_NPs was 82% beginning from 10 up to 120 µg/mL. The adsorption performed at pH value 7 for 30 mL of the adsorbent solution stirred for 120 min. While CuFe_2_O_4_/PANI nano-composites exhibit higher removal efficiency of 99.5% when varying the concentration of Hg^2+^ from 10 to 32 µg/mL. It begins to decrease to 92.3% upon increasing the concentration of mercury up to 120 µg/mL (Figure 4).

#### 2.2.2. Effect of Contact Time

**Figure 5:**
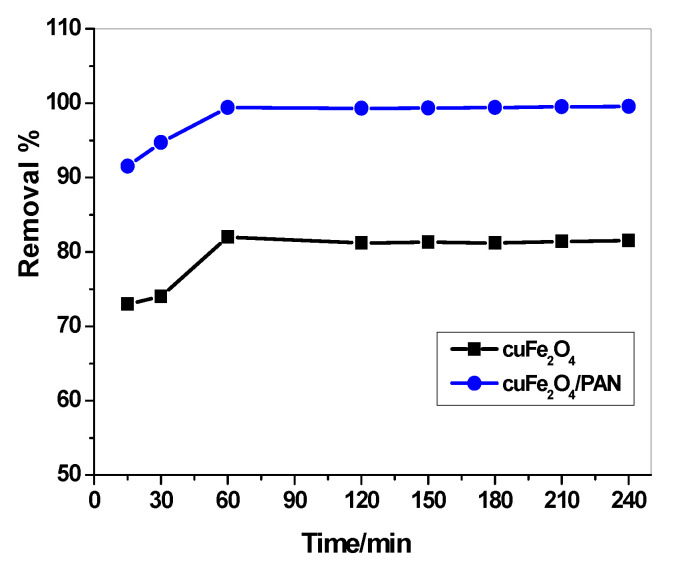
Effect of contact time on adsorption optimization using CuFe_2_O_4_ and CuFe_2_O_4_/PANI nano-composites sorbents (V = 30 mL, Hg^2+^ concentration = 25 µg/mL, adsorbent amount = 0.1 g CuFe_2_O_4_ and 0.2 g CuFe_2_O_4_/PANI and pH 7).

As shown in Figure 5, the concentration of Hg(II) ions was studied relative to the contact time of each adsorbent. It was found that the time required to obtain more than 80% of Hg(II) removal was 2 h for CuFe_2_O_4_. However, in case of CuFe_2_O_4_/PANI composite, the time required to achieve the equilibrium was one hour with a removal percentage of 99.5%. To examine the adsorption mechanism, kinetics is the vital feature. Pseudo first order and second order models were fitted as the practical kinetics data. The obtained results were presented in Table 2. The adsorption process for both CuFe_2_O_4_ and CuFe_2_O_4_/PANI composite obeyed the second order model.

#### 2.2.3. Effect of Adsorbent Amount

**Figure 6:**
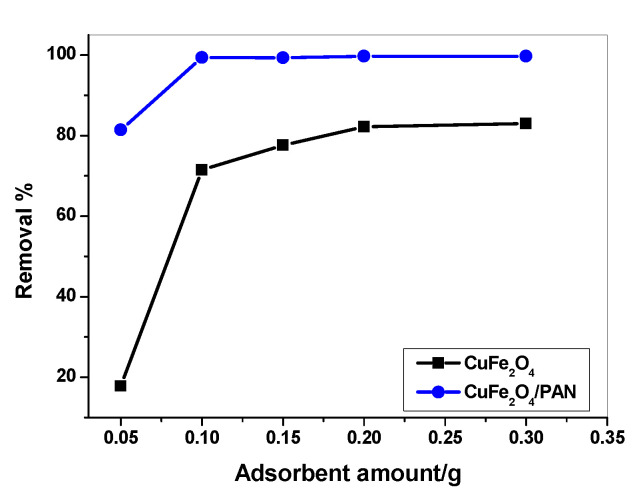
Effect of CuFe_2_O_4_ and CuFe_2_O_4_/PANI nano-composite sorbents amount on adsorption optimization (V = 30 mL, Hg^2+^ concentration = 25 µg/mL, contact time =2 h for CuFe_2_O_4_ and 1 h for CuFe_2_O_4_/PANI and pH 7).

To optimize the amount of adsorbent NPs, different amounts from each adsorbent in the range of 0.05 to 0.3 g were put in contact with 30 mL of 25 µg/mL Hg^2+^ solutions of pH 7 and 60 min contact time. As shown in Figure 6, it was observed that the maximum adsorption (i.e., 99.5% removal efficiency) was attained after using 0.2 and 0.1 g for CuFe_2_O_4_NPs and CuFe_2_O_4_/PANI composite, respectively.

#### 2.2.4. Effect of pH

**Figure 7:**
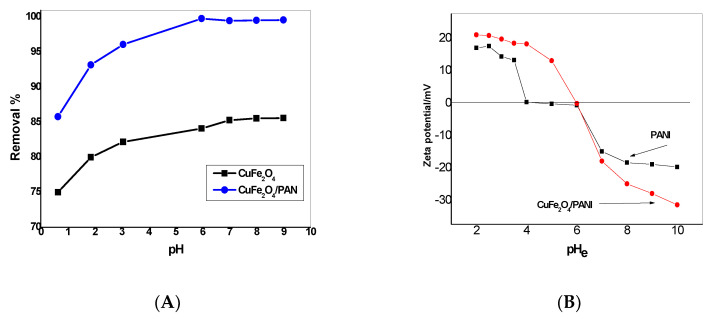
(**A**) Effect of pH on adsorption optimization using CuFe_2_O_4_ and CuFe_2_O_4_/PANI nano-composite sorbents (V= 30 mL, Hg^2+^ concentration= 25 µg/mL, adsorbent amount = 0.1 g CuFe_2_O_4_ and 0.2 g CuFe_2_O_4_/PANI and contact time = 2 h for CuFe_2_O_4_ and 1 h for CuFe_2_O_4_/PANI). (**B**) Plots of the zeta potential as a function of pH for CuFe_2_O_4_/PANI and PANI.

The pH is an essential parameter for Hg^2+^ adsorption due to its relevance to Hg speciation, as well as the interactions between Hg species and adsorbent surfaces. When the feed water pH was varied from 6.0 to 9.0, Hg^2+^ removal efficiency of CuFe_2_O_4_/polyaniline remained at ~99.5% (Figure 7A). For CuFe_2_O_4_NPs the removal percentage of Hg(II) ions became constant until pH reaches 7. This can be explained that at higher pH values, oxygen-containing groups (e.g., –OH) are ionized to –O–, forming negative charges on the CuFe_2_O_4_ surface.

Based on zeta potential results (Figure 7B),the Point of Zero Charge for both PANI and CuFe_2_O_4_/PANI composite, is around 4–6 and 6, respectively. CuFe_2_O_4_/PANI composite had net negative charges at pH > 6.0 and positive charges at pH < 6.0. At low pH values (e.g., pH < 5.0 for PANI-HCl), nitrogen atoms of imine groups were preferentially bound by protons, causing the PANI surfaces carrying positive charges.

### 2.3. Adsorption Isotherms

**Figure 8:**
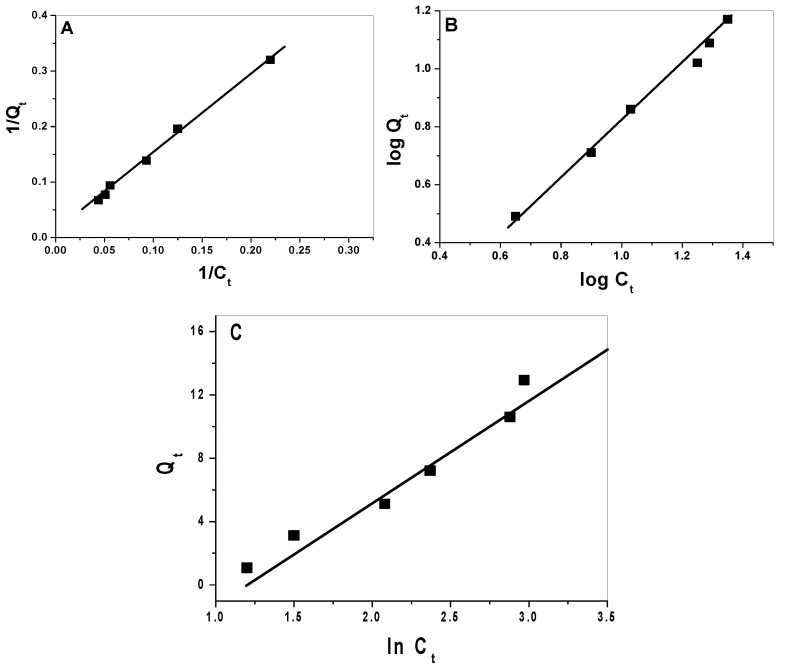
(**A**) Langmuir, (**B**) Freundlich, and (**C**) Temkin isotherms for mercury removal using CuFe_2_O_4_ nano-particles (NPs).

**Figure 9:**
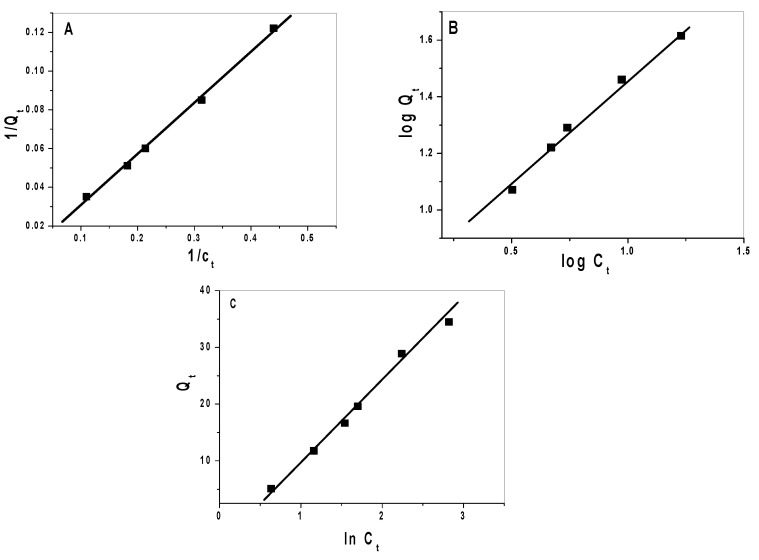
(**A**) Langmuir, (**B**) Freundlich, and (**C**) Temkin isotherms for mercury removal using CuFe_2_O_4_/PANi nano-composite.

Langmuir (Equation (2), Freundlich (Equation (3), and Temkin (Equation (4) models were applied to calculate the sorption of Hg^2+^ ions for both CuFe_2_O_4_ and CuFe_2_O_4_/PANI nano-composite (Figure 8 and Figure 9).

1/*Q_t_* = 1/*X_m_b C_t_* + 1/*X_m_* (2)

*Log Q_t_* = (1/*n*) *log C_t_* + *log k_F_* (3)

*Q_t_* = (*RT*/*B_T_*) *ln C_t_* + (*RT*/*B_T_*) *ln K_T_* (4)

where: *Q_t_* is adsorption capacity at equilibrium (mg/g), *C_t_* is equilibrium concentration of the Hg^2+^ solution (µg/mL), *t* (min) is contact time, *X_m_* (mg/g) is maximum monolayer adsorption capacity and *b* (L/mg) is the adsorption equilibrium constant. Relative adsorption capacities and sorption intensities *n* and *K_f_* (mg/g), and the constants of Freundlich model, were calculated. Temkin constants, *B_T_* (kJ/mol) and *K_T_* (L/mg) whose are constants of heat of sorption and maximum binding energy were estimated. A 30 mL of different mercury concentrations ranging from 10 to 200 µg/mLwere tested under the optimum conditions and the adsorption was expressed by three equilibrium models: Langmuir, Freundlich, and Temkin to illustrate the adsorption capacity and adsorption behavior. The theory of Langmuir assumes that the adsorption occurs by monolayer on the surface of the adsorbent with the same adsorption sites (homogeneous surface), while Freundlich is an empirical theory at which the adsorption occurs by multilayer on the surface of the adsorbent with different adsorption sites (heterogeneous surface). Temkin assumed that there are indirect interactions between adsorbate molecules and the heat of adsorption of all molecules decrease linearly with increasing surface coverage [44]. The results are summarized in Table 3 and confirm the reasonable adsorption capacity of the used nano-composite material and follows Langmuir isotherm model.

### 2.4. Competitive Adsorption of Different Heavy Metals

**Figure 10:**
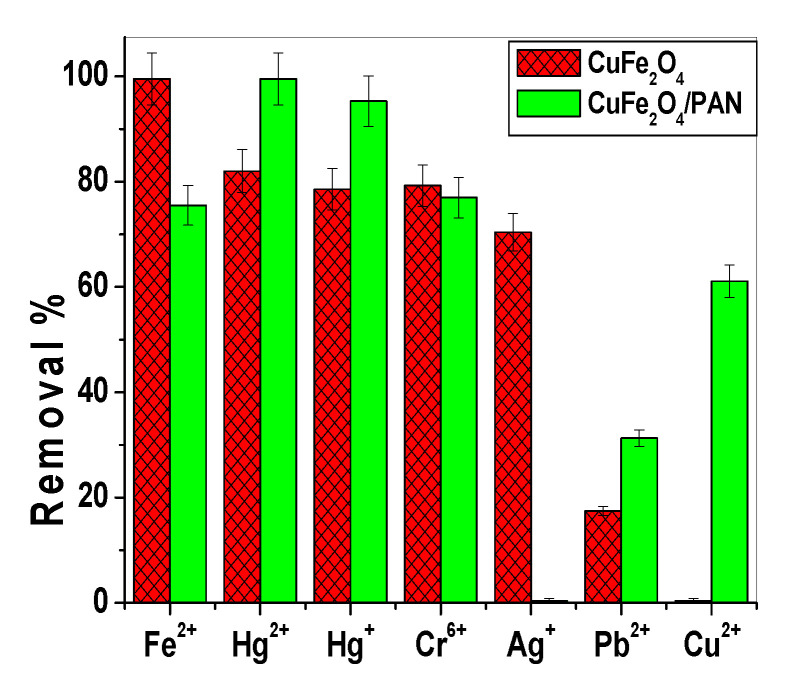
Removal of different metal ions with CuFe_2_O_4_ and CuFe_2_O_4_/PANI nano-composite.

The adsorption of some metal ions such as Hg^+^, Hg^2+^, Fe^2+^, Cu^2+^, Cr^6+^, Pb^2+^, and Ag^+^ was investigated. CuFe_2_O_4_NPs revealed an affinity order: Fe^2+^ > Hg^2+^ > Hg^+^ ~ Cr^6+^ > Ag^+^ >> Pb^2+^. No remarkable adsorption for Cu^2+^ ions using CuFe_2_O_4_NPs. For CuFe_2_O_4_/PANI nano-composite, the affinity order was: Hg^2+^ > Hg^+^ > Fe^2+^~ Cr^6+^ > Cu^2+^ > Pb^2+^. No remarkable adsorption for Ag^+^ ions using CuFe_2_O_4_/PANI nano-composite. The removal percentage of the studied ions using CuFe_2_O_4_NPs and CuFe_2_O_4_/PANI nano-composite is shown in Figure 10. From the mentioned results, CuFe_2_O_4_/PANI nano-composite revealed an enhanced removal power towards inorganic mercury than CuFe_2_O_4_NPs only.

### 2.5. Regeneration

**Figure 11:**
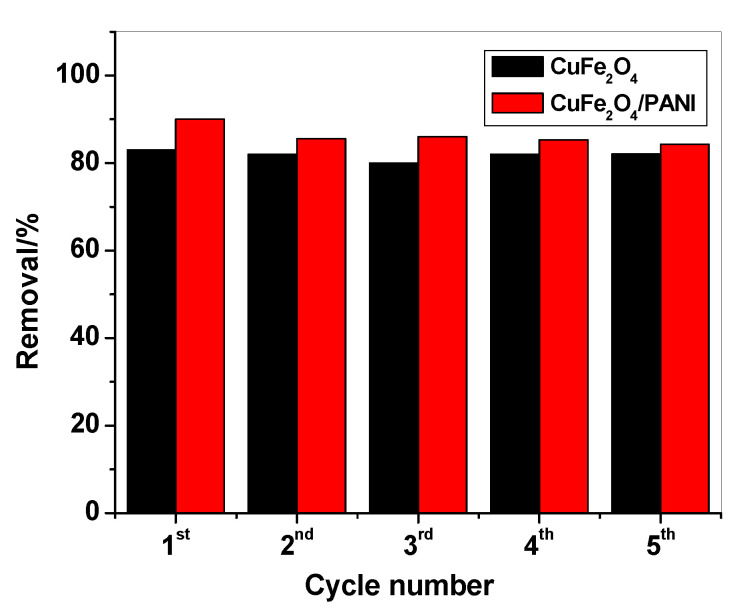
Mercury removal by CuFe_2_O_4_ and CuFe_2_O_4_/PANI nano-composites after regeneration.

The adsorbent material was regenerated after each adsorption cycle of mercury by washing with 0.1 M acetic acid. After five cycles of regeneration, the efficiency of CuFe_2_O_4_ NPs for the removal of Hg^+^ ions remains 82.0% however, there was a decrease in the removal efficiency of the CuFe_2_O_4_/PANI sorbent reached to 85.3% as shown in Figure 11.

### 2.6. Comparison with Other Sorbents for Mercury Removal

Water pollution becomes a critical issue around the world, and heavy metals contribute to major pollution in water. The application of nano-materials for the removal of mercuric ions from water has attracted significant attention. Table 4 summarizes some of reported sorbents used for mercury removal included the present work. The prepared copper ferrite loaded by polyaniline provides higher maximum adsorption capacity [12,17,18,20,26], short contact time [12,20,23,27], andhigher removal percentage [12,18,19,20,21,24,25,26,27].

### 2.7. Mechanism of Adsorption

**Figure 12:**
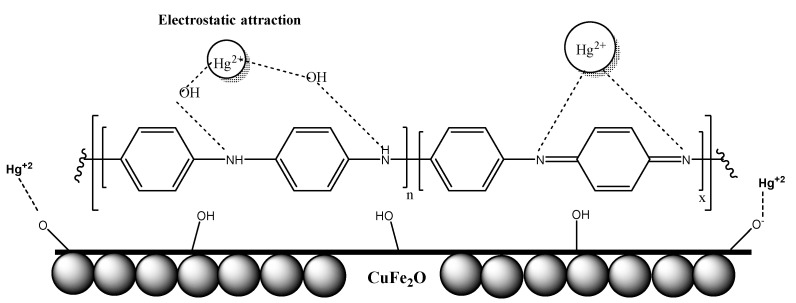
Schematic of Hg^2+^ adsorption mechanism.

The adsorption mechanism of Hg^2+^ ions using CuFe_2_O_4_ and CuFe_2_O_4_/PANI composite is shown in Figure 12. The adsorption mechanism can be explained in two ways. Physical adsorption can be occurred on the surface of PANI layer or in the porosity of the adsorbent or chemical adsorption through the interaction between the PANI base layers with mercuric ions. In addition, at the working pH value, oxygen-containing groups (e.g., –OH) in CuFe_2_O_4_ can be ionized to –O^−^, forming negative charges on the CuFe_2_O_4_ surface and enhance the favorable adsorption of Hg^2+^ ions.

## 3. Materials and Methods

### 3.1. Materials

For the experimental purpose, all chemicals used were of analytical reagent grade, 98–99%. Metal nitrates, chloride, and sulfate were of the highest purity and supplied by Sigma-Aldrich (St. Louis, MO, USA). Polyethylene glycol 6000 (PEG), potassium hydroxide, and ammonium peroxydisulfate were purchased from Fluka (Ronkonoma, NY, USA). Aniline was purchased from Central Drug House Ltd. (New Delhi, India) and distilled prior to use. All the chemicals were used as received without any further purification.

### 3.2. Apparatus

High-resolution transmission electron microscopy (HRTEM) images were taken by JEOL-JEM-2100 electron microscope instrument (Osaka, Japan). The prepared adsorbents were characterized by X-ray diffraction (XRD) which were carried out by BRUKER D2 PHASER 2nd generation X-ray diffractometer (Berline, Germany) using CuK*α*,*β* radiation (λ = 0.154 nm) in the angular region of 2*θ* = 4–80°. Operation conditions were 40KV, 40 mA and scanning speed of 8°/min. The Brunauer–Emmett–Teller (BET) surface area measurements were carried out by N_2_ adsorption–desorption at 77 K using Nova 3200 s (Florida, FL, USA) unite instrument, in the relative pressure (*p/p^o^)* at 0.25104. Fourier Transform Infrared (ATR-FTIR) was used to obtain the spectra in a spectral range of 4000–500 cm^−1^. Inductively coupled argon plasma (ICAP 6500 Duo, Thermo Scientific, Abingdon, UK) as used for mercury ion evaluation.

### 3.3. Preparation of CuFe_2_O_4_Nano-Particles

The synthesis of the nano-particles was done by using the co-precipitation technique [45]. Briefly 11.7 mmol CuSO_4_ and 14.98 mmol FeCl_3_ were dissolved in 200 mL 1 wt.% PEG solution. The solution was kept under stirring for about one hour to insure the equilibrium between all the components. To the above mixture, 4M KOH was added drop-wise with vigorous stirring until reaching a pH 9. The mixture was kept under magnetic stirring for another two hours then aged overnight. The precipitate was filtered, washed with distilled water until it was free from Cl^−^ and SO_4_^2−^ ions and dried at 70 °C for two h. The precipitated was then calcined at 600 °C in air for 3 h and then ground using agate motor to obtain a fine powder.

### 3.4. Preparation of CuFe_2_O_4_/PANI Nano-Composite

The polyaniline copper ferrite nano-composite was prepared using chemical polymerization method by dispersing 2 g of the previously prepared CuFe_2_O_4_ nano-particles in 200 mL of 2M HCl and stirred vigorously at room temperature for 10 min. A 4.5 mL aliquot of distilled aniline monomer was added under continuous stirring for 30 min. To the above suspension, 20 mL of 19.7 mmol (NH_4_)_2_S_2_O_8_ solution was added drop-wisely, as a polymerization initiator. An immediate color change of the solution to blue green was observed. The suspension was stirred to complete the polymerization process for about 1 h. The copper ferrite doped PANI was separated on a filter paper, rinsed with distilled water, and finally dried at 100°C in an electrical oven. The produced powder has a green color which represents emeraldine salt of polyaniline.

### 3.5. Removal of Mercury from Waste Water

A range of mercury (II) ion (10–120 µg/mL) was prepared. For the adsorption studies different amounts of either CuFe_2_O_4_ or CuFe_2_O_4_/PANI nano-composite (ranged from 0.05 to 0.3 g) were added to 30 mL of the prepared solution at room temperature and pH 7. These solutions were stirred for a contact time varied from 15 min to 2 h. After adsorption, the solutions were filtered and the adsorbent material was separated. The concentration of Hg^2+^ ion was evaluated before and after the removal of mercury by inductively coupled argon plasma.

The removal percentage of mercury was calculated using the following equation:

*Removal*% = ((*C*_0_ − *C_t_*))/*C*_0_× 100 (5)

where, *C*_0_ and *C_t_* are the mercury concentration in µg/mL at initial and after time *t*, respectively.

## 4. Conclusions

CuFe_2_O_4_/PANI nano-composite was successfully prepared and its adsorption properties towards Hg^2+^ ions removal were checked. An X-ray diffractometer, TEM, and BET were used to characterize the prepared nano-composites. The crystallite size of the synthesized CuFe_2_O_4_ and CuFe_2_O_4_/PANI nano-composite was 10.2 and 23.4 nm, respectively. Under the optimum conditions, CuFe_2_O_4_/PANI offer higher removal efficiency than CuFe_2_O_4_ for Hg^+^/ Hg^+2^ ions which were 95.3 and 99.5%, respectively. Both adsorbents followed the second order model and Langmuir model with adsorption capacity of 12.5 and 157.1 mg/g for CuFe_2_O_4_ and CuFe_2_O_4_/PANI composite, respectively. After five cycles of regeneration, the efficiency of CuFe_2_O_4_NPs for the removal of Hg^+^ ions remains 82.0% however, there was a decrease in the removal efficiency of the CuFe_2_O_4_/PANI sorbent reached to 85.3%with lower efficiency and good performance when used again after five cycles. These materials were successfully applied for the removal of Hg^2+^ ions with a high efficiency over other studied heavy metals.

## Figures and Tables

**Table 1:** General surface characteristics of CuFe_2_O_4_ and CuFe_2_O_4_/PANI nano-composites obtained by N_2_ adsorption at 77 K.

Sample	Surface Area(m^2^/g)	Average Pore Volume(cm^3^/g)	Average Pore Diameter (nm)
CuFe_2_O_4_/PANI	30.8	0.06	17.8
CuFe_2_O_4_ NP	44.7	0.11	9.9

**Table 2:** Adsorption kinetics parameters.

Adsorbent	Pseudo-First Order			Second Order			
	k_1_(min^−1^)	q_e1_(mg/g)	R^2^	k^2^(g/(mg. min))	q_e2_(mg/g)	q_e_^exp^	R^2^
**CuFe_2_O_4_**	0.0056	1.571	0.942	5.3 × 10^−3^	5.8922	7.1086	0.991
**CuFe_2_O_4_/PANI**	0.0732	2.2134	0.953	0.1121	8.3356	8.4123	0.998

**Table 3:** Isotherm constants for the adsorption of mercury onto CuFe_2_O_4_ and CuFe_2_O_4_/PANI nano-composites.

Model	Parameters	CuFe_2_O_4_	CuFe_2_O_4_/PANI	Unit
Langmuir	X_m_	12.5	157.1	mg/g
	B	0.561	0.153	L/mg
	R^2^	0.998	0.999	
Freundlich	N	1.06	1.34	mg/g
	K_f_	2.75	5.24	mg/g
	R^2^	0.997	0.995	
Temkin	K_T_	0.34	0.744	L/mg
	b_T_	0.371	0.162	K J/mol
	R^2^	0.980	0.997	

**Table 4:** Some materials used for the removal of mercuric ion.

Adsorbent Type	Maximum Adsorption Capacity mg/g	Contact Time	Removal %	Ref.
Poly(vinylalcohol)/poly(vinylimidazole) complexing membrane	120	125 min	99.4	[12]
Dithiocarbamate-incorporated mono size polystyrene	33.2	30 min	NR	[17]
Magnetic iron oxide nanoparticles modified with 2-mercaptobenzothiazole	0.59	4 min	98.6	[18]
Thiolated multi-walled carbon nanotubes	204.64	40 min	98	[19]
Amidoamine functionalized multi-walled carbon nanotubes (MWCNT-AA)	101.35	180 min	80	[20]
Mercaptopropyl-coated cobalt ferrite (CoFe_2_O_4_) magnetic nanoparticles	NR	30 min	97	[21]
Poly(aniline-co-5-sulfo-2-anisidine) nanoparticles	2063	48 h	99.8	[23]
Gold Nanoparticle−Aluminum Oxide	676	30 min	>97	[24]
Mercaptoamine-functionalised silica-coated magnetic nanoparticles (MAF-SCMNPs)	355	120 min	NR	[25]
Polyaniline Nanotubes	0.8239	60 min	90	[26]
Iron oxide nanoparticles	NR	24 h	87	[27]
CuFe_2_O_4_ CuFe_2_O_4_/PAN	12.5 157.1	120 min 60 min	82 99.5	This work

## Supplementary Materials

The following are available online, Figure S1: FTIR spectra of (A) CuFe_2_O_4_ and (B) CuFe_2_O_4_/PANI nanocomposites; Figure S2: Thermal-gravimetric analysis (TGA) of CuFe_2_O_4_/PANI.
