# Supplementary material for: CuFe2O4/Polyaniline (PANI) Nanocomposite for the Hazard Mercuric Ion Removal: Synthesis, Characterization, and Adsorption Properties Study
Source: Molecules. 2020 Jun 12;25(12):2721. doi: 10.3390/molecules25122721 (PMC7356621; doi:10.3390/molecules25122721)
Supplement: Supplementary file 1 [file molecules-25-02721-s001.pdf]

Supplementary file

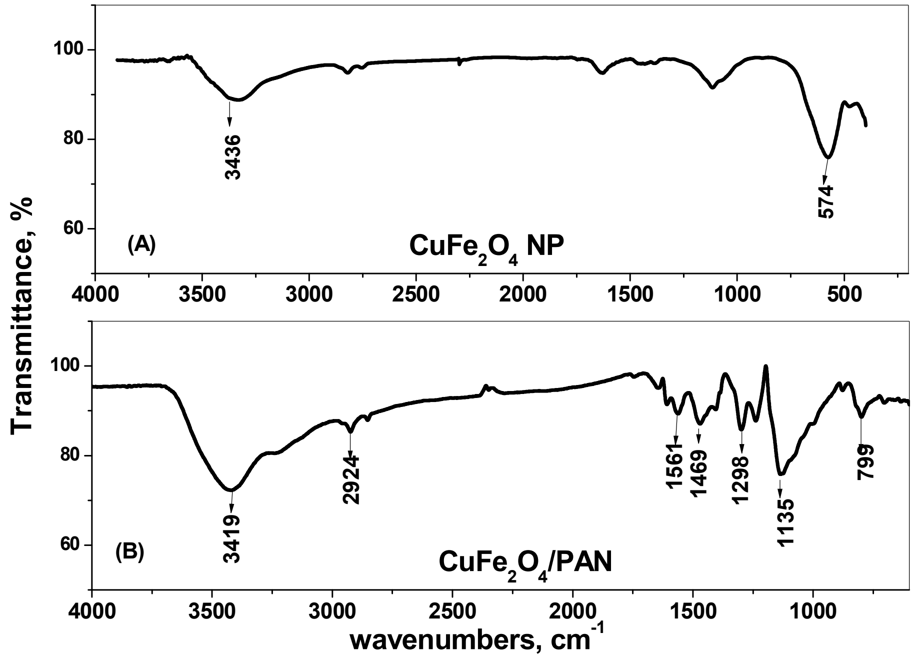

Figure S1. FTIR spectra of (A) $CuFe_2O_4$ and (B) $CuFe_2O_4$/PANI nanocomposites.

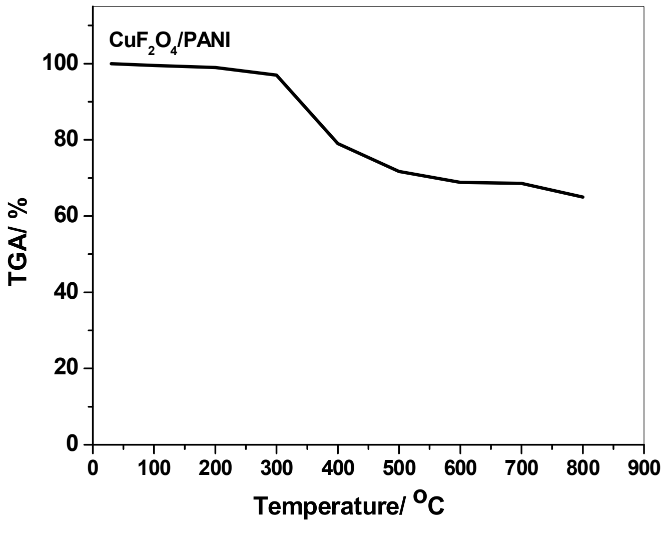

Figure S2. Thermal-gravimetric analysis (TGA) of $CuFe_2O_4$/PANI
